# Microbiological Profile of Instrumented Spinal Infections: 10-Year Study at a French Spine Center

**DOI:** 10.3390/antibiotics13090791

**Published:** 2024-08-23

**Authors:** Sophie Reissier, Carine Couzigou, Romain Courseau, Elise Aubert, Alban Le Monnier, Eric Bonnet, Peter Upex, Pierre-Emmanuel Moreau, Guillaume Riouallon, Julie Lourtet-Hascoët

**Affiliations:** 1 Laboratoire de Microbiologie Clinique et Plateforme de Dosage des Anti-Infectieux, Groupe Hospitalier Paris Saint Joseph, 75014 Paris, France; elise.aubt@gmail.com (E.A.); alemonnier@ghpsj.fr (A.L.M.); julielourtet@hotmail.com (J.L.-H.); 2 Laboratoire de Bactériologie-Hygiène Hospitalière, CHU de Rennes, 35033 Rennes, France; 3 Équipe Mobile de Microbiologie Clinique, Groupe Hospitalier Paris Saint Joseph, 75014 Paris, France; ccouzigou@ghpsj.fr; 4 Service de Chirurgie Orthopédique, Groupe Hospitalier Paris Saint Joseph, 75014 Paris, France; rcourseau@ghpsj.fr (R.C.); pupex@ghpsj.fr (P.U.); pemoreau@ghpsj.fr (P.-E.M.); griouallon@ghpsj.fr (G.R.); 5 Équipe Mobile d’Infectiologie, Hôpital Joseph Ducuing, 31300 Toulouse, France; drmaboule1711@gmail.com

**Keywords:** surgical site infection, spinal instrumentation, Enterobacterales

## Abstract

Objective: The objective was to compare the microbiological characteristics and treatment of early and late surgical site infections (SSIs) in instrumented spinal surgery. Methods: Those patients admitted for SSIs in a single center between January 2010 and December 2022 were included. The subjects were divided into early (eSSIs) and late (lSSIs) SSIs, and demographic, microbiological, treatment, and follow-up data were collected. Results: Instrumented spinal surgery was performed in 2136 patients. Ninety-six cases of infections were identified (prevalence = 4.5%), with 47.9% eSSIs and 52.1% lSSIs. In 58.7% of the cases, the eSSIs were monomicrobial: *Staphylococcus aureus* (37%) and Enterobacterales (33.3%) were the main bacteria involved. In 66% of the cases, the lSSIs, were monomicrobial: *Cutibacterium acnes* (30.3%) and staphylococci were predominant. Enterobacterales were isolated in more than 70% of the polymicrobial samples in both the eSSIs and lSSIs. The treatment of the eSSIs mostly consisted of lavage-debridement surgery associated with antibiotic treatment, while the treatment of the lSSIs combined hardware removal or replacement and long-duration antibiotic treatment. A negative outcome was observed in 17.1% of the eSSIs and 5.7% of the lSSIs. Enterobacterales were associated with negative outcomes of eSSIs. Conclusions: Enterobacterales were found in most of the polymicrobial infections regardless of the time of infection onset. Further large studies should be conducted to precisely determine the management and prevention regarding the increasing Gram-negative bacteria SSIs.

## 1. Introduction

Spinal instrumentation is frequently required in the cases of fracture, spinal deformation, or degenerative disorders. The postoperative complications include surgical site infection (SSI), which occurs in one to fourteen percent of the cases after spine instrumentation depending on the studies and the population reviewed [1,2,3,4]. The risk factors for infection include the patient’s characteristics, microbiological factors, and the type of surgical procedure [3,5,6]. The use of instrumentation also has an impact on the occurrence of postoperative infections. It increases local inflammation, facilitating bacterial adherence and biofilm formation [7]. Patients with a wound infection have longer hospital stays, higher mortality, and the risk of repeated surgical interventions. These aspects increase the cost of care [8]. SSIs represent a diagnostic and therapeutic challenge. Few studies have reported the microbiological epidemiology of SSIs. They showed that *Staphylococcus aureus*, Enterobacterales, and Coagulase-negative staphylococci were the main microorganisms responsible for SSIs, but these results vary from one study to another [9,10,11,12].

The aim of this study was to compare the microbiological characteristics of SSIs after spinal instrumentation according to their time of onset and their impact on the management.

## 2. Results

Spine surgery with instrumentation was performed in 2136 patients between 2010 and 2022. The prevalence of SSIs was 4.5% (n = 96). Early SSIs (eSSIs) accounted for 47.9% (n = 46) and late SSIs (lSSIs) for 52.1% (n = 50) of the cases.

### 2.1. Study Population

Forty-six patients were treated for an eSSI (twenty-six men and twenty women). The median age was 50.5 years [17–89 years], and the median delay between surgery and infection was 17 days [1–30 days]. Nearly 39% (n = 18) of the patients had at least one comorbidity, including a history of spine surgery (n = 15, 83.3%) and diabetes (n = 7, 38.9%) (Table 1). Fifty patients were treated for lSSIs (thirty-four men and sixteen women). The median age was 51.7 years [15–87 years], and the median delay between surgery and infection was 8 months [31 days–21 years]. Forty-two percent (n = 21) of the patients reported at least one comorbidity. Thirteen patients (61.9%) had a history of spinal deformity due to scoliosis or cerebral palsy (Table 1). Most of the surgical procedures were performed by the posterior approach in both groups (94% and 96.9% in the eSSI and lSSI groups, respectively).

### 2.2. Microbiological Findings

**Figure 1:**
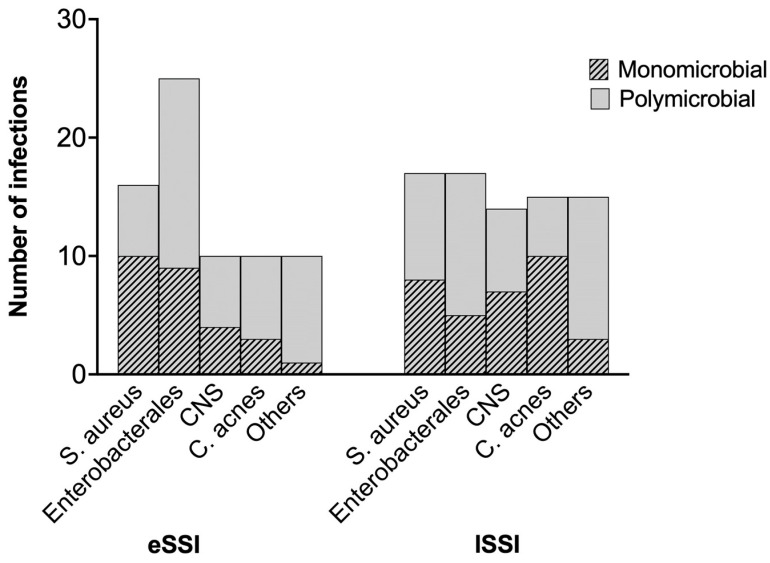
Bacteria species identified in eSSIs and lSSIs. CNS: Coagulase-negative staphylococci. “Others” refers to all bacteria described in Table 1 and Table 2 other than *S. aureus*, Enterobacteriaceae, *C. acnes*, and CNS.

The bacteria species identified in eSSIs and lSSIs are represented in Figure 1. Enterobacterales were identified in 54.3% and 34% of the eSSIs and lSSIs, respectively, and were significantly more involved in the eSSIs (*p* = 0.04). *S. aureus* accounted for almost 35% of the bacteria identified in both groups. The prevalence of Coagulase-negative staphylococci (CNS)-positive samples in the eSSI group was equivalent to that of *Cutibacterium acnes*-positive samples, at 22%. In lSSIs, CNS and *C. acnes* were both involved in 30% of the infections.

#### 2.2.1. Early Infections (n = 46)

The infection was monomicrobial in 58.7% (n = 27) of the cases. *S. aureus* was the main pathogen involved in the eSSIs (n = 10, 37%), followed by Enterobacterales (*Escherichia coli*, *Klebsiella oxytoca*, *Enterobacter cloacae,* and *Proteus mirabilis*) (n = 9, 33.3%). *E. coli* was the most isolated Enterobacterale. CNSs were responsible for the infection in four patients (14.8%). In the polymicrobial infections, which accounted for 41.3% (n = 19), Enterobacterales and bacteria from skin flora (CNS and *C. acnes*) were the main pathogens. They were identified in 16 (84.2%) and 13 (68.4%) samples, respectively. As in monomicrobial infections, *E. coli* was the most frequent Enterobacterale. *S. aureus* was involved in nearly a third of the polymicrobial eSSIs (n = 6, 31.6%). Finally, streptococci and enterococci were involved in 26.3% of the infections (Table 2; Figure 1).

**Table 2:** Microbiological characteristics of early SSIs.

	Monomicrobial (n = 27) (%)	Polymicrobial (n = 19) (%)	Total (n = 46) (%)
*Staphylococcus aureus*	10 (37)	6 (31.6)	16 (31.6)
*Escherichia coli*	5 (18.5)	6 (31.6)	11 (23.9)
*Cutibacterium acnes*	3 (11.1)	7 (36.8)	10 (21.7)
*Staphylococcus epidermidis*	3 (11.1)	5 (26.3)	8 (17.8)
*Proteus mirabilis*	1 (3.7)	4 (21.1)	5 (10.9)
*Klebsiella pneumoniae*	-	3 (15.8)	3 (6.5)
*Klebsiella oxytoca*	1 (3.7)	1 (5.3)	2 (4.3)
*Enterobacter cloacae*	2 (7.4)	-	2 (4.3)
*Pseudomonas aeruginosa*	-	2 (10.5)	2 (4.3)
*Enterococcus faecalis*	-	3 (15.8)	2 (4.3)
*Staphylococcus capitis*	1 (3.7)	-	1 (2.2)
*Streptococcus anginosus*	-	2 (10.5)	2 (4.3)
*Bacteroides fragilis*	-	2 (10.5)	2 (4.3)
*Citrobacter koseri*	-	1 (5.3)	1 (2.2)
*Staphylococcus saccharolyticus*	-	1 (5.3)	1 (2.2)
*Corynebacterium striatum*	1 (3.7)	-	1 (2.2)

For polymicrobial infections, the (%) corresponds to the number of samples in which the bacterium was isolated out of the total number of polymicrobial samples. In total column, the (%) corresponds to the number of samples in which the bacterium was isolated out of the total number of infections.

#### 2.2.2. Late Infections (n = 50)

The majority of the lSSIs were monomicrobial (66%, n = 33). *C. acnes* was the predominant pathogen in the monomicrobial lSSIs (n = 10, 30.3%), followed by *S. aureus* and CNS (n = 8, 24.2%; n = 7, 21.2%, respectively). Enterobacterales, mostly *E. coli,* were responsible for five infections (15.2%). Seventeen infections (34%) were polymicrobial, and Enterobacterales were isolated in over 70% of the samples (n = 12). Seven different species of Enterobacterales were identified, the most common being *E. coli*. Bacteria belonging to skin flora (CNS, *Corynebacterium* spp., and *C. acnes*) were associated with most of the polymicrobial infections (n = 15, 88.2%). *S. aureus* was identified in more than half of the positive samples (n = 9, 52.9%). Streptococci and enterococci were involved in 23.5% of the infections (n = 4). Finally, anaerobic bacteria were isolated in three cases (17.6%) (Table 3; Figure 1).

### 2.3. Surgical and Antibiotic Treatment

#### 2.3.1. Early Infections (n = 46)

Surgical and antibiotic treatment information was available for 42 patients. The treatments mostly consisted of debridement, antibiotic treatment, and implant retention (DAIR) (n = 32, 78%). All the patients for whom the treatment information was available received a combination of two antibiotics (n = 42, 100%). The empirical treatment was based on a combination of vancomycin or daptomycin and a broad-spectrum beta-lactam (ceftriaxone, cefepime, or piperacillin–tazobactam). Antibiotic therapy was adapted after a few days according to the microbiological results. Treatment was continued orally for an average total duration of 6 weeks.

#### 2.3.2. Late Infections

The data on surgical management were available for forty-seven patients. In 90% of the cases (n = 45), surgery was performed, consisting of surgical debridement without hardware removal for twenty-three (51.1%) patients and with hardware removal for twenty-two (48.9%) patients. Of these twenty-two patients, seven underwent hardware replacement at the same time. Surgical treatment was associated with antibiotic therapy for all the patients, for an average duration of 9 weeks. Two patients were treated with antibiotics only.

### 2.4. Follow-Up

In the eSSI group, follow-up data were available for forty-one patients. Seven patients had a negative outcome (17.1%): two died from causes unrelated to the infection and five had a relapse of the infection, which was polymicrobial in all the cases and involved *E. coli* in four cases.

In the lSSI group, follow-up data were available for thirty-three patients. Two relapses were observed (5.7%), involving polymicrobial infections and bacteria from the skin flora.

## 3. Discussion

In this retrospective study, we report the clinical and microbiological characteristics of early and delayed spinal implant infections. The two studied populations had relatively similar demographic characteristics. In the eSSI group, the median delay between hardware implantation and infection was 17.3 days. The infection occurred in patients with a median age of 50.5 years with a previous history of spine surgery, diabetes, scoliosis, cerebral palsy, or severe immunodepression. The most frequent history aspect was spinal surgery, observed in 55.6% of the patients. Some previous studies described an increased risk of infection in the case of a previous surgery, especially in the spinopelvic area [13,14]. In previous studies, diabetes and obesity were also identified as perioperative risk factors for SSIs [15,16,17]. In our study, diabetes was noted in 25.9% of the patients with eSSIs. In the lSSI group, the patients’ characteristics were similar, with a median age of 51.7 years and 42% of the patients with at least one comorbidity. The most common comorbidity was also a spine surgery history. The patients included in our study were younger than those in most of the published data (trauma, patients with cerebral palsy), but, according to Peng et al., age does not appear to be a risk factor for SSIs [3].

The microbiological findings in eSSIs showed that 58.7% of the monomicrobial infections with *S. aureus* were predominantly isolated, which is consistent with previous studies [1,10,12,18]. Surprisingly, we observed a significantly higher prevalence of Enterobacterales in eSSIs compared to lSSIs.

In the monomicrobial samples from eSSIs, Enterobacterales accounted for 33% of the bacteria involved, whereas the prevalence described in the literature varied widely from study to study but was less than 30% [1,10,12,18,19]. In polymicrobial infections, we also observed a very high prevalence of Enterobacterales (84.2%). A high proportion of Enterobacterale-related infections may be explained by several factors: a local contamination in the case of a high inoculum caused by genitourinary or fecal incontinence, the posterior surgical approach, or extended instrumentation localized in the lumbar or lower thoracic spine [2,20,21]. Moreover, more patients with eSSIs were treated for trauma, and previous studies have reported that Gram-negative bacteria may be found in cases of infections with spinal material placed for traumatic causes [22,23]. In 2014, Nunez-Pereira et al. described a significant association between urinary tract infections and SSIs caused by the same microorganism, mostly Enterobacterales [19]. Unfortunately, a history of urinary tract infections was not investigated in our study.

In the lSSI group, we observed a lower but nonetheless significant rate of polymicrobial infections, with a high rate of samples positive for Gram-negative bacilli. These results were consistent with previous published studies: Dubee et al. found 44% of Enterobacterales and 34% of polymicrobial infections and Mok et al. found 56.3% of polymicrobial infections. In our study, nearly 30% of the patients had cerebral palsy with significant motor disability, which probably has a major impact on perineal bacteria colonization and may be linked to their SSI. The local inoculum of bacteria, especially for Gram-negative bacteria and anaerobes, is increased and may play a role in the occurrence of these lSSIs [24]. Several sources for late infections are reported in previous studies. Late-onset infections may be caused by the hematogenous seeding of bacteria or a late exacerbation of low-virulent bacteria that are part of the cutaneous flora [23,24]. *C. acnes* was the microorganism most involved in monomicrobial lSSIs in our study, accounting for 30.3% of the patients. *C. acnes* is frequently reported in lSSIs, especially in posterior spinal instrumentation and in the case of non-union [25,26,27,28]. The incidence of *C. acnes* lSSIs was higher in our study, probably because of the improvement in the culture media for these slow-growing bacteria.

The surgical management of spinal infections differs significantly according to the time of infection. In eSSIs, the surgical strategy is DAIR, like the management of prosthetic joint infections. In lSSIs, bacteria may coat the prosthetic material with a polysaccharide matrix, or biofilm, which limits the antibiotic penetration and boosts the pathogen virulence. The treatment guidelines recommend surgery with the removal of the infected material associated with the antibiotics penetrating within the biofilm. The antibiotic treatment used in lSSIs was chosen according to the antibiotic susceptibilities and biofilm diffusion ability. It consisted of the combination of fluoroquinolones (levofloxacin or moxifloxacin) and rifampicin on susceptible strains and clindamycin, tetracyclines, or sulfamethoxazole in the case of fluoroquinolone-resistant strains.

Most of the patients with eSSIs (78%) were managed with the DAIR procedure regardless of the microbiology results according to the national and international guidelines. Our data support the conclusions of previous studies that eSSIs should preferentially be managed by the DAIR procedure [10,13,24,29].

By contrast, in the lSSI group, 49.9% of the patients underwent surgery with hardware removal or replacement. Removing material from spinal instrumentation is complex and increases the risk of infection, especially with large instrumentation. The DAIR procedure may be the first option in this case in order to also limit the risk of surgical infection. A long-duration antibiotic therapy (6 and 9 weeks on average, respectively) was associated with surgery in all the patients, in accordance with the bone and joint recommendations [6,30,31].

We reported relapse rates of 17.1% in eSSIs and 5.1% in lSSIs. In the eSSI relapses, almost all the relapses occurred in infections caused by Gram-negative bacteria or polymicrobial infections involving Gram-negative bacteria. Wille et al. also reported an association between Gram-negative bacteria infections and the risk of relapse [23]. Infections due to Gram-negative bacteria are increasingly reported, and the management of these infections remains difficult [32,33]. In our study, these infections occurred in patients with several comorbidities.

The bacteria reported in early or late infections showed variable epidemiology. In our study, we found a high number of Gram-negative bacteria and staphylococci in both the eSSI and lSSI groups, which led us to propose a combination of daptomycin or vancomycin and a broad-spectrum beta-lactam (cefepim or tazocillin) as an empirical treatment for all the patients.

Regarding the oral treatment, antibiotics should be adapted to the microbiological documentation. The surgical sites may be close to the skin flora (staphylococci or *C. acnes*) or flora containing Gram-negative bacteria (lumbar or sacral area); it is relevant to take these results into account when selecting an antibiotic therapy. However, these results are found in patients managed in a single-center spine department, so they should be adapted according to the local epidemiology and antimicrobial resistances. Our findings are in accordance with the antibiotic treatment protocols depending on the time of infection occurrence and with the French recommendations regarding disco-vertebral infections’ diagnosis and management that were recently published [34,35].

This study presents bias related to the monocentric retrospective study design: the lack of information about the long-term follow-up, the postoperative outcomes, and antibiotic treatments. A prospective multicentric study is necessary to confirm our data.

## 4. Materials and Methods

A retrospective and descriptive study was conducted at Saint-Joseph hospital (Paris, France) and was based on data collected from Medical Information Systems Program (MISP) correlated with the microbiological laboratory database. The study protocol was approved by the local ethics committee. All patients over 15 years admitted to the Spine Center of the Orthopedic Department for SSIs between January 2010 and December 2022 were included in the study.

### 4.1. Patients and Samples

Demographic data (age, gender, and medical history) were collected for each patient. Comorbidities were defined as severe immunodeficiency (cancer or autoimmune disease), history of spinal surgery, history of scoliosis, cerebral palsy, and diabetes. Diagnosis of SSIs was based on clinical, biological, microbiological, and radiological arguments, according to French guidelines published in 2023 [35]. “Early” infections (eSSIs) were defined as infections occurring less than 30 days after surgery. “Late” infections (lSSIs) were defined as infections occurring 30 days or more after surgery. Microbiological diagnosis was performed on three to five deep intraoperative samples for each patient. Appropriate culture media were used, and incubation times were extended to identify aerobic, anaerobic, and slow-growing bacteria. For each surgical site, solid and tissue specimens were collected in sterile vials containing stainless beads and articular fluids were inoculated in blood culture bottles. Gram staining was performed for each sample on day one. Solid specimens were then crushed by vortexing for 10 min in 1 mL of saline solution. Standard cultures were performed on Columbia blood agar, chocolate agar PolyViteX, and schaedler solution (bioMérieux, Marcy-l’Etoile, France). All samples were incubated with CO_2_ and under anaerobic atmosphere for 15 days. Media were observed daily for microbial growth. In case of positive culture, identification was performed by MALDITOF-MS (Andromas Software, LT2-Andromas, Paris, France). Antimicrobial susceptibilities were tested by disk diffusion method on solid Mueller–Hinton agar media (bioMérieux, Marcy-l’Etoile, France) according to the French and European recommendations of the Committee of Antibiotic Susceptibility of Microbiology [36,37]. Microbiological diagnosis of SSI was established if at least one intraoperative sample culture identified bacteria that did not belong to the cutaneous flora. For bacteria of cutaneous flora (CNS, *C. acnes*, and *Corynebacterium* spp.), diagnosis of infection was retained if bacteria were identified in two or more intraoperative samples with identical antibiotic susceptibility profiles. Results are presented to show SSI patients including all bacteria found, with details of monomicrobial and polymicrobial infections (at least two bacteria species found in deep samples).

### 4.2. Treatment and Follow-Up

All patients were managed in the Orthopedic Department by a multidisciplinary team, which included an orthopedic surgeon, an infectious diseases specialist, a radiologist, and a microbiologist. Empirical intravenous antibiotic treatment combining vancomycin and piperacillin–tazobactam or cefepime was prescribed and secondarily adapted to microbiological results. An oral antibiotic treatment was planned for at least four weeks after intravenous treatment, according to French guidelines. Patients’ outcomes were based on at least one-year follow-up. They were evaluated with clinical, biological, and radiological examinations. A favorable outcome was considered when good clinical recovery, satisfactory joint mobility, and no sign of inflammation were observed. Failure was defined by relapse of infection.

### 4.3. Statistical Analysis

Data were analyzed using GraphPad Prism 9 (GraphPad Software, Inc., La Jolla, CA, USA). Patient age and time between initial surgery and infection were expressed by the minimum, maximum, and median. The group comparisons of the analyzed distributions were carried out by Fisher’s exact test and Chi^2^ test, with a threshold of 5% bilateral.

## Figures and Tables

**Table 1:** Demographics and characteristics of patients included according to infection occurrence.

	eSSI (n = 46) (%)	lSSI (n = 50) (%)	*p* Value
Gender			
Men	26 (56.5)	34 (68)	0.29
Women	20 (43.5)	16 (32)	0.29
Age (median/years)	50.5	51.7	0.73
Delay surgery/infection (median/days)	17.3	245.5	
Comorbidities	27 (58.7)	21 (42)	0.66
Spine surgery	15 (55.6)	14 (66.7)	0.55
Immunodeficiency	4 (14.8)	4 (19)	0.72
Diabetes	7 (25.9)	2 (9.5)	0.26
Scoliosis	6 (22.2)	7 (33.3)	0.52
Cerebral palsy	8 (29.6)	6 (28.6)	1
Infection			
Monomicrobial	27 (58.7)	33 (66)	0.46
Polymicrobial	19 (41.3)	17 (33)	0.46
Surgical approach			
ND	12 (26.1)	18 (36)	
Posterior	32 (94.1)	31 (96.9)	1
Median	2 (5.9)	1 (3.1)	1
Surgical treatment	41 (97.6)	45 (95.7)	
ND	4 (8.7)	3 (6)	
DAIR	32 (78)	23 (51.1)	<0.001
Hardware removal	2 (4.9)	15 (33.3)	<0.001
Hardware replacement	7 (17.1)	7 (15.6)	1
Antibiotic therapy	42 (91.3)	48 (96)	1
Duration (mean/weeks)	6	9	

ND: not documented; DAIR: debridement, antibiotic treatment, and implant retention.

**Table 3:** Microbiological characteristics of late SSIs.

	Monomicrobial (n = 33) (%)	Polymicrobial (n = 17) (%)	Total (n = 50) (%)
*Staphylococcus aureus*	8 (24.2)	9 (52.9)	17 (34)
*Cutibacterium acnes*	10 (30.3)	5 (29.4)	15 (30)
*Staphylococcus epidermidis*	5 (15.2)	5 (29.4)	10 (20)
*Escherichia coli*	3 (9.1)	4 (23.4)	7 (14)
*Staphylococcus lugdunensis*	1 (3)	2 (11.8)	3 (6)
*Corynebacterium* spp.	-	3 (17.6)	3 (6)
*Proteus mirabilis*	1 (3)	2 (11.8)	3 (6)
*Enterococcus faecalis*	2 (6.1)	1 (5.9)	3 (6)
*Serratia marcescens*	1 (3)	1 (5.9)	2 (4)
*Morganella morganii*	-	2 (11.8)	2 (4)
*Streptococcus agalactiae*	-	2 (11.8)	2 (4)
*Bacteroides fragilis*	-	2 (11.8)	2 (4)
*Enterobacter cloacae*	-	1 (5.9)	1 (2)
*Staphylococcus capitis*	1 (3)	-	1 (2)
*Cutibacterium avidum*	1 (3)	-	1 (2)
*Citrobacter koseri*	-	1 (5.9)	1 (2)
*Klebsiella aerogenes*	-	1 (5.9)	1 (2)
*Pseudomonas aeruginosa*	-	1 (5.9)	1 (2)
*Streptococcus pyogenes*	-	1 (5.9)	1 (2)
*Peptoniphilus indolicus*	-	1 (5.9)	1 (2)

For polymicrobial infections, the (%) corresponds to the number of samples in which the bacterium was isolated out of the total number of polymicrobial samples.

## Data Availability

Data are contained within the article.

## References

[1] Al Farii H., Slawaska-Eng D., Pankovitch S., Navarro-Ramirez R., Weber M. (2021). Gram-Negative Surgical Site Infections after 989 Spinal Fusion Procedures: Associated Factors and the Role of Gram-Negative Prophylactic Antibiotic Coverage. Int. J. Spine Surg..

[2] Pull ter Gunne A.F., van Laarhoven C.J.H.M., Cohen D.B. (2010). Incidence of Surgical Site Infection Following Adult Spinal Deformity Surgery: An Analysis of Patient Risk. Eur. Spine J..

[3] Peng X.-Q., Sun C.-G., Fei Z.-G., Zhou Q.-J. (2019). Risk Factors for Surgical Site Infection after Spinal Surgery: A Systematic Review and Meta-Analysis Based on Twenty-Seven Studies. World Neurosurg..

[4] Abdul-Jabbar A., Takemoto S., Weber M.H., Hu S.S., Mummaneni P.V., Deviren V., Ames C.P., Chou D., Weinstein P.R., Burch S. (2012). Surgical Site Infection in Spinal Surgery: Description of Surgical and Patient-Based Risk Factors for Postoperative Infection Using Administrative Claims Data. Spine.

[5] Fang A., Hu S.S., Endres N., Bradford D.S. (2005). Risk Factors for Infection after Spinal Surgery. Spine.

[6] Dubée V., Leflon-Guibout V., Lenoir T., Fantin B. (2012). Les Infections Du Site Opératoire Après Chirurgie Rachidienne Instrumentée. J. Anti-Infect..

[7] Schierholz J.M., Beuth J. (2001). Implant Infections: A Haven for Opportunistic Bacteria. J. Hosp. Infect..

[8] Radcliff K.E., Neusner A.D., Millhouse P.W., Harrop J.D., Kepler C.K., Rasouli M.R., Albert T.J., Vaccaro A.R. (2015). What Is New in the Diagnosis and Prevention of Spine Surgical Site Infections. Spine J..

[9] Kohler P., Eshaghi A., Kim H.C., Plevneshi A., Green K., Willey B.M., McGeer A., Patel S.N. (2018). Toronto Invasive Bacterial Diseases Network (TIBDN) Prevalence of Vancomycin-Variable Enterococcus Faecium (VVE) among vanA-Positive Sterile Site Isolates and Patient Factors Associated with VVE Bacteremia. PLoS ONE.

[10] Kowalski T.J., Berbari E.F., Huddleston P.M., Steckelberg J.M., Mandrekar J.N., Osmon D.R. (2007). The Management and Outcome of Spinal Implant Infections: Contemporary Retrospective Cohort Study. Clin. Infect. Dis..

[11] Shillingford J.N., Laratta J.L., Reddy H., Ha A., Lehman R.A., Lenke L.G., Fischer C.R. (2018). Postoperative Surgical Site Infection after Spine Surgery: An Update from the Scoliosis Research Society (SRS) Morbidity and Mortality Database. Spine Deform..

[12] Rico Nieto A., Loeches Yagüe B., Quiles Melero I., Talavera Buedo G., Pizones J., Fernández-Baillo Sacristana N. (2024). Descriptive Study of Spinal Instrumentation-Related Infections in a Tertiary Hospital. Rev. Esp. Cir. Ortop. Traumatol..

[13] Gerometta A., Olaverri J.C.R., Bitan F. (2012). Infections in Spinal Instrumentation. Int. Orthop..

[14] Cizik A.M., Lee M.J., Martin B.I., Bransford R.J., Bellabarba C., Chapman J.R., Mirza S.K. (2012). Using the Spine Surgical Invasiveness Index to Identify Risk of Surgical Site Infection: A Multivariate Analysis. J. Bone Jt. Surg. Am..

[15] Olsen M.A., Nepple J.J., Riew K.D., Lenke L.G., Bridwell K.H., Mayfield J., Fraser V.J. (2008). Risk Factors for Surgical Site Infection Following Orthopaedic Spinal Operations. J. Bone Jt. Surg. Am..

[16] Epstein N.E. (2007). Do Silver-Impregnated Dressings Limit Infections after Lumbar Laminectomy with Instrumented Fusion?. Surg. Neurol..

[17] Friedman N.D., Sexton D.J., Connelly S.M., Kaye K.S. (2007). Risk Factors for Surgical Site Infection Complicating Laminectomy. Infect. Control Hosp. Epidemiol..

[18] Köder K., Hardt S., Gellert M.S., Haupenthal J., Renz N., Putzier M., Perka C., Trampuz A. (2020). Outcome of Spinal Implant-Associated Infections Treated with or without Biofilm-Active Antibiotics: Results from a 10-Year Cohort Study. Infection.

[19] Núñez-Pereira S., Rodríguez-Pardo D., Pellisé F., Pigrau C., Bagó J., Villanueva C., Cáceres E. (2014). Postoperative Urinary Tract Infection and Surgical Site Infection in Instrumented Spinal Surgery: Is There a Link?. Clin. Microbiol. Infect..

[20] Mok J.M., Cloyd J.M., Bradford D.S., Hu S.S., Deviren V., Smith J.A., Tay B., Berven S.H. (2009). Reoperation after Primary Fusion for Adult Spinal Deformity: Rate, Reason, and Timing. Spine.

[21] Cahill P.J., Warnick D.E., Lee M.J., Gaughan J., Vogel L.E., Hammerberg K.W., Sturm P.F. (2010). Infection after Spinal Fusion for Pediatric Spinal Deformity: Thirty Years of Experience at a Single Institution. Spine.

[22] Hedge A., Mohan S., Lim W.E.H. (2012). Infections of the Deep Neck Spaces. Singap. Med. J..

[23] Wille H., Dauchy F.-A., Desclaux A., Dutronc H., Vareil M.-O., Dubois V., Vital J.-M., Dupon M. (2017). Efficacy of Debridement, Antibiotic Therapy and Implant Retention within Three Months during Postoperative Instrumented Spine Infections. Infect. Dis..

[24] Fernandez-Gerlinger M.-P., Arvieu R., Lebeaux D., Rouis K., Guigui P., Mainardi J.-L., Bouyer B. (2019). Successful 6-Week Antibiotic Treatment for Early Surgical-Site Infections in Spinal Surgery. Clin. Infect. Dis..

[25] Clark C.E., Shufflebarger H.L. (1999). Late-Developing Infection in Instrumented Idiopathic Scoliosis. Spine.

[26] Schofferman L., Zucherman J., Schofferman J., Hsu K., Gunthorpe H., Picetti G., Goldthwaite N., White A. (1991). Diptheroids and Associated Infections as a Cause of Failed Instrument Stabilization Procedures in the Lumbar Spine. Spine.

[27] Bémer P., Corvec S., Tariel S., Asseray N., Boutoille D., Langlois C., Tequi B., Drugeon H., Passuti N., Touchais S. (2008). Significance of Propionibacterium Acnes-Positive Samples in Spinal Instrumentation. Spine.

[28] Shifflett G.D., Bjerke-Kroll B.T., Nwachukwu B.U., Kueper J., Burket J., Sama A.A., Girardi F.P., Cammisa F.P., Hughes A.P. (2016). Microbiologic Profile of Infections in Presumed Aseptic Revision Spine Surgery. Eur. Spine J..

[29] Bosch-Nicolau P., Rodríguez-Pardo D., Pigrau C., Pellisé F., Haddad S., Lung M., Almirante B. (2019). Acute Spinal Implant Infection Treated with Debridement: Does Extended Antibiotic Treatment Improve the Prognosis?. Eur. J. Clin. Microbiol. Infect. Dis..

[30] Viola R.W., King H.A., Adler S.M., Wilson C.B. (1997). Delayed Infection after Elective Spinal Instrumentation and Fusion. A Retrospective Analysis of Eight Cases. Spine.

[31] Richards B.R., Emara K.M. (2001). Delayed Infections after Posterior TSRH Spinal Instrumentation for Idiopathic Scoliosis: Revisited. Spine.

[32] Choi J.H.-K., Duong H.A., Williams S., Lee J., Oh M., Rosen C., Lee Y.-P., Bhatia N. (2021). The Efficacy of Bactrim in Reducing Surgical Site Infections after Spine Surgery. N. Am. Spine Soc. J..

[33] Long D.R., Bryson-Cahn C., Pergamit R., Tavolaro C., Saigal R., Chan J.D., Lynch J.B. (2021). 2021 Young Investigator Award Winner: Anatomic Gradients in the Microbiology of Spinal Fusion Surgical Site Infection and Resistance to Surgical Antimicrobial Prophylaxis. Spine.

[34] Triffault-Fillit C., Ferry T., Laurent F., Pradat P., Dupieux C., Conrad A., Becker A., Lustig S., Fessy M.H., Chidiac C. (2019). Microbiologic Epidemiology Depending on Time to Occurrence of Prosthetic Joint Infection: A Prospective Cohort Study. Clin. Microbiol. Infect..

[35] Lacasse M., Derolez S., Bonnet E., Amelot A., Bouyer B., Carlier R., Coiffier G., Cottier J.P., Dinh A., Maldonado I. (2023). 2022 SPILF–Clinical Practice Guidelines for the Diagnosis and Treatment of Disco-Vertebral Infection in Adults. Infect. Dis. Now.

[36] CASFM Recommandations 2013–2021. https://www.sfm-microbiologie.org.

[37] Eucast: Breakpoint Tables for 2013–2021. https://www.eucast.org.

